# Supplementary material for: Abcc6 Null Mice—a Model for Mineralization Disorder PXE Shows Vertebral Osteopenia Without Enhanced Intervertebral Disc Calcification With Aging
Source: Front Cell Dev Biol. 2022 Feb 3;10:823249. doi: 10.3389/fcell.2022.823249 (PMC8850990; doi:10.3389/fcell.2022.823249)
Supplement: Supplementary file 1 [file DataSheet1.pdf]

Supplementary Material

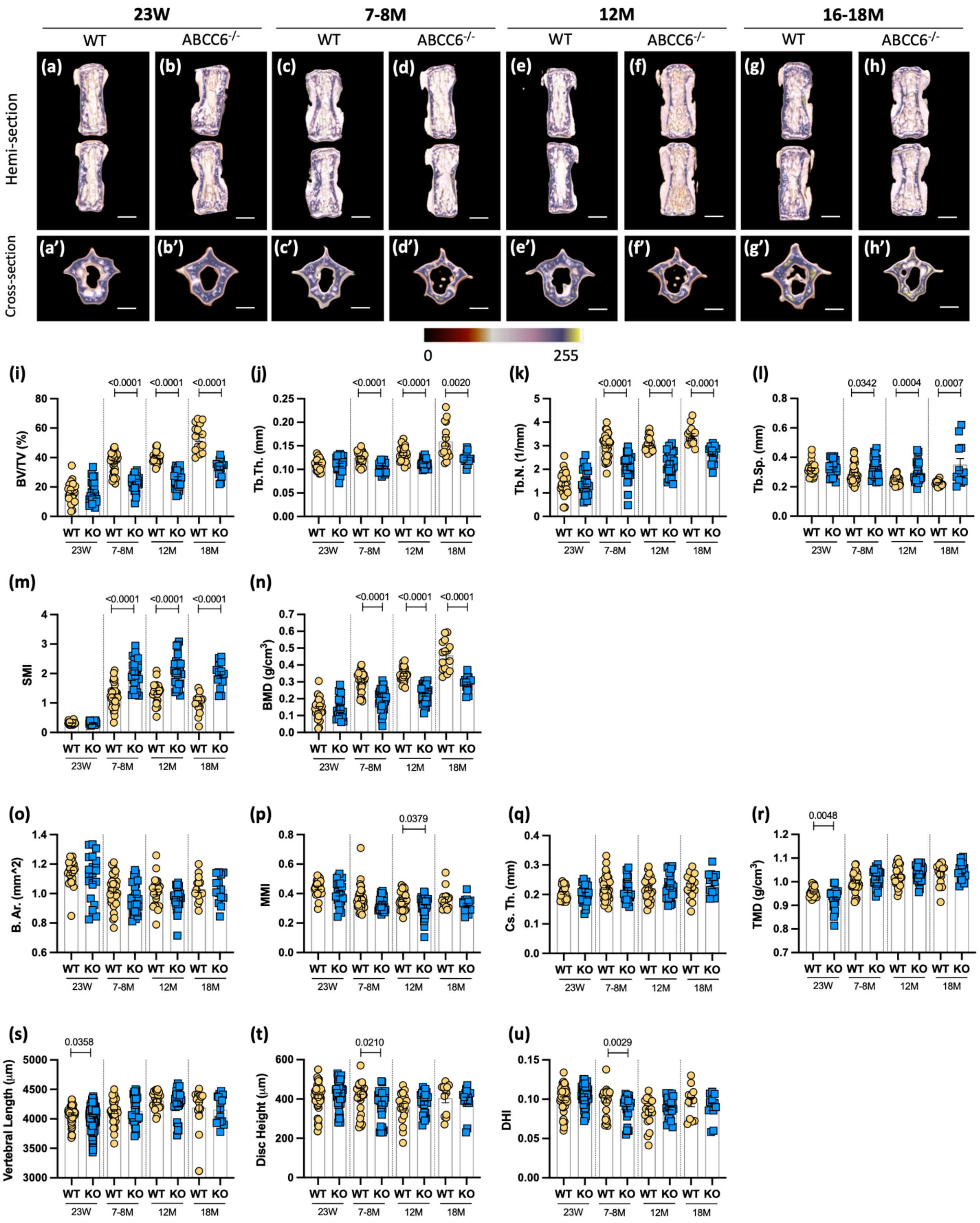
23W
7-8M
12M
16-18M
WT
ABCC6-/-
Hemi-section
Cross-section
(a) (b) (c) (d) (e) (f) (g) (h)
(a') (b') (c') (d') (e') (f') (g') (h')
0
255
(i) BV/TV (%)
<0.0001
(j) Tb.Th. (mm)
0.0020
(k) Tb.N. (1/mm)
(l) Tb.Sp. (mm)
0.0342
0.0004
0.0007
(m) SMI
(n) BMD (g/cm3)
(o) B. Ar. (mm^2)
(p) MMI
0.0379
(q) Cs. Th. (mm)
(r) TMD (g/cm3)
0.0048
(s) Vertebral Length (μm)
0.0358
(t) Disc Height (μm)
0.0210
(u) DHI
0.0029
WT KO
23W 7-8M 12M 18M

Supplementary Figure 1. *Abcc6*$^{-/-}$ mice exhibit trabecular thinning of caudal vertebrae. Representative microCT reconstructions of *(a-h)* hemi- and *(a'-h')* cross-sections show consistent trabecular thinning in caudal vertebrae of *Abcc6*$^{-/-}$ mice at all timepoints. Quantitative microCT analysis of trabecular bone *(i-n)* BV/TV, Tb.Th., Tb.N., Tb.Sp., SMI, BMD, and cortical bone *(o-r)* B.Ar., MMi, Cs.Th., TMD. *(s)* Vertebral length, *(t)* disc height and *(u)* DHI are shown for caudal motion segments. Quantitative analyses are shown as mean ±SD (n = 2-4 discs/mice; 3-5 vertebrae/mice, n ≥ 5 mice/genotype). Significance was determined using unpaired t-test or Mann Whitney as appropriate. Scale bar=1 mm. BV/TV= bone volume/tissue volume. Tb.Th.= trabecular thickness. Tb.N.= trabecular number. Tb.Sp.= trabecular spacing. SMI= structural model index. BMD= bone mineral density. B.Ar.= bone area. MMI= mean polar moment of inertia. Cs.Th.= cross-sectional thickness. DHI= disc height index.

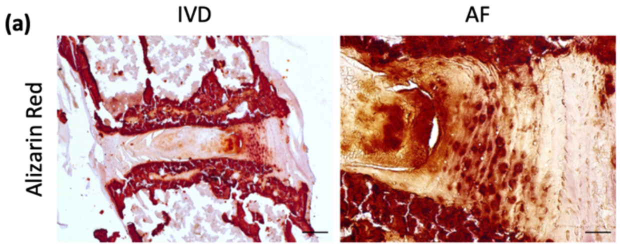

Supplementary Figure 2. Presence of robust Alizarin Red staining within AF of 16-18M *Abcc6*$^{-/-}$ disc. *(a)* Representative Alizarin Red staining showing robust free calcium staining within the AF region of a 16-18M *Abcc6*$^{-/-}$ mouse disc.

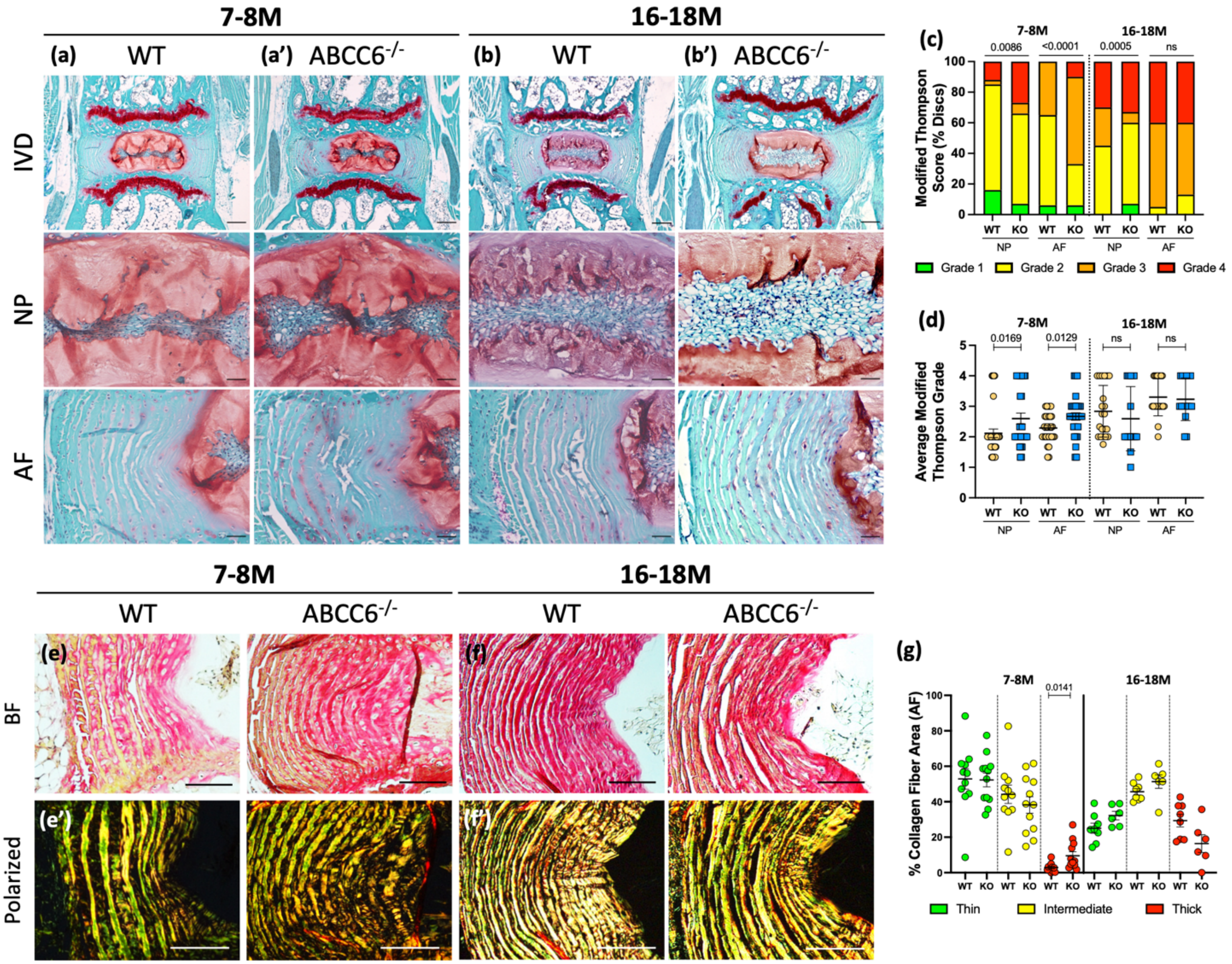

Supplementary Figure 3. Caudal discs of *Abcc6*$^{-/-}$ mice showed minor AF degeneration with comparable collagen fiber composition. *(a-b’)* Safranin O/Fast Green and hematoxylin staining of *(a-a’)* 7-8 month and *(b-b’)* 16–18-month-old lumbar discs showed tissue morphology and proteoglycan content consistent with age-related disc degeneration (row 1, scale bar = 200µm and rows 2-3, scale bar = 50µm). *(c, d)* Histological grading analysis using the modified Thompson scale showed altered levels of NP and AF degradation but similar average Thompson grades. *(e-g)* Picrosirius Red staining and quantification of percent collagen fiber area showed no difference in overall collagen fiber thickness in the AF of *Abcc6*$^{-/-}$ caudal discs. Quantitative analyses are shown as mean ±SD. Significance of grading distribution was determined using a x^2 test. Significance of average grade data and percent area were determined using unpaired t-test or Mann Whitney test as appropriate.

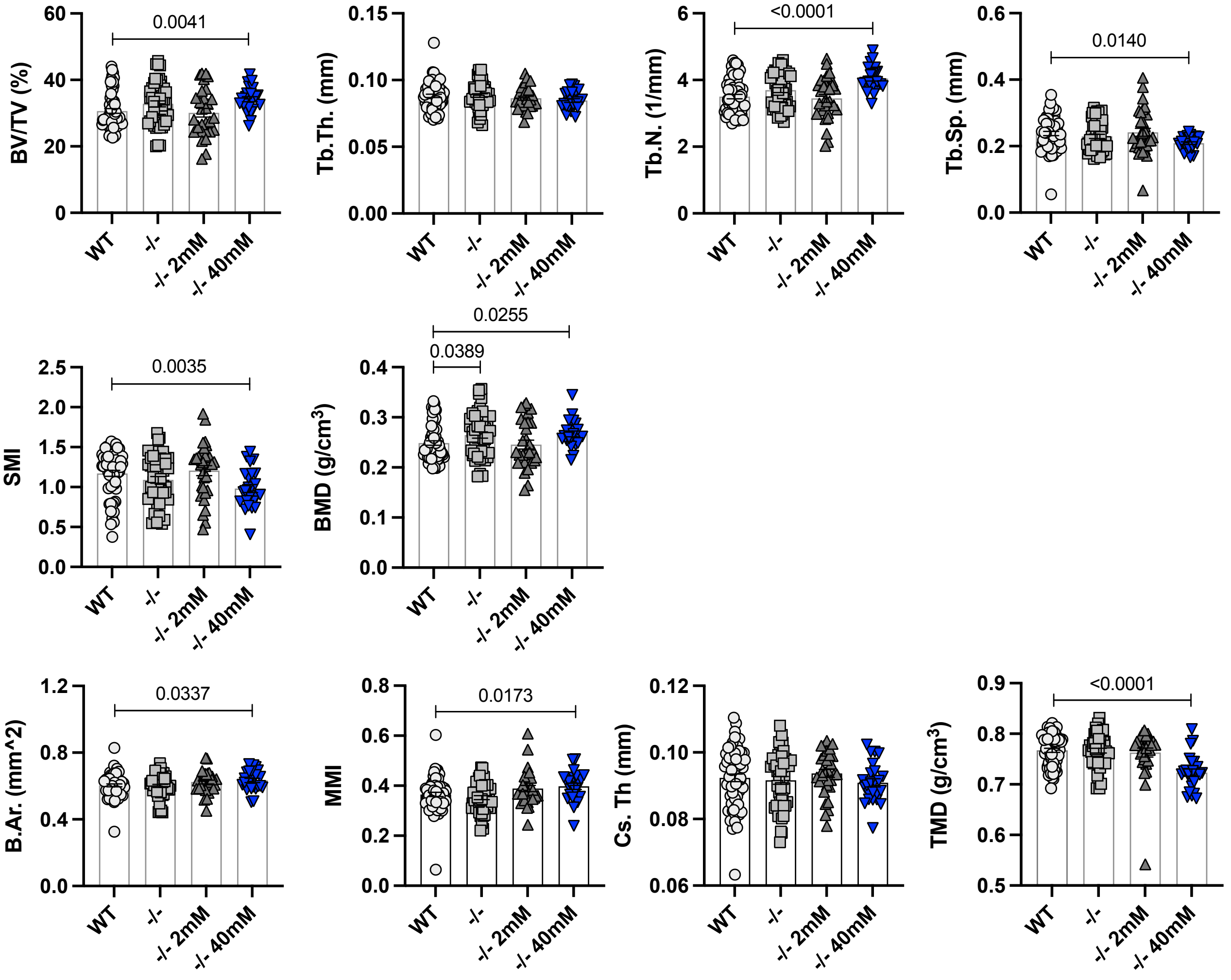

Supplementary Figure 4. Effect of K3Citrate treatment on vertebral bone quality in *Abcc6*$^{-/-}$ mice. Quantitative microCT analysis of trabecular bone parameters *(a-f)* BV/TV, Tb.Th., Tb.N., Tb.Sp., SMI, BMD, and cortical bone parameters *(g-j)* B.Ar., MMi, Cs.Th., TMD of 23-week-old WT and *Abcc6*$^{-/-}$ mice treated with or without 2 mM and 40 mM K3Citrate for 20 weeks. Quantitative analyses are shown as mean ±SD (n = 4 vertebrae/mouse, n ≥ 5 mice/genotype). Significance was determined using unpaired t-test or Mann Whitney as appropriate. BV/TV= bone volume/tissue volume. Tb.Th.= trabecular thickness. Tb.N.= trabecular number. Tb.Sp.= trabecular spacing. SMI = structural model index. BMD = bone mineral density. B.Ar.= bone area. MMI= mean polar moment of inertia. Cs.Th.= cross-sectional thickness.
